# Computation of Nonlinear Parameters of Heart Rhythm Using Short Time ECG Segments

**DOI:** 10.1155/2015/983479

**Published:** 2015-01-22

**Authors:** Berik Koichubekov, Ilya Korshukov, Nazgul Omarbekova, Viktor Riklefs, Marina Sorokina, Xenia Mkhitaryan

**Affiliations:** Department of Medical Biophysics and Informatics, Karaganda State Medical University, Gogol Street 40, Karaganda 100008, Kazakhstan

## Abstract

We propose the method to compute the nonlinear parameters of heart rhythm (correlation dimension *D*_2_ and correlation entropy *K*_2_) using 5-minute ECG recordings preferred for screening of population. Conversion of RR intervals' time series into continuous function *x*(*t*) allows getting the new time series with different sampling rate *dt*. It has been shown that for all *dt* (250, 200, 125, and 100 ms) the cross-plots of *D*_2_ and *K*_2_ against embedding dimension *m* for phase-space reconstruction start to level off at *m* = 9. The sample size *N* at different sampling rates varied from 1200 at *dt* = 250 ms to 3000 at *dt* = 100 ms. Along with, the *D*_2_ and *K*_2_ means were not statistically different; that is, the sampling rate did not influence the results. We tested the feasibility of the method in two models: nonlinear heart rhythm dynamics in different states of autonomous nervous system and age-related characteristics of nonlinear parameters. According to the acquired data, the heart rhythm is more complex in childhood and adolescence with more influential parasympathetic influence against the background of elevated activity of sympathetic autonomous nervous system.

## 1. Background

Heart rate variability (HRV) is a noninvasive measurement of cardiovascular autonomic regulation. Specifically, HRV is a measurement of the interaction between sympathetic and parasympathetic activity in autonomic functioning. The analysis of heart rate variability is based mainly on analysis of RR intervals. RR intervals are the series of time intervals between heartbeats. There are two main approaches for analysis: time domain analysis of HRV and frequency domain analysis. Recent results on HRV signal analysis show that its dynamic behavior involves nonlinear components that also contribute in the signal generation and control. There are publications thoroughly elaborating the effectiveness of linear and nonlinear techniques in analyzing heart rate variability [1–3]. Many authors agree that nonlinear methods complement the linear ones. In some cases, these methods may uncover hidden abnormalities or alterations in heart rate and have prognostic value. Previous studies have assessed gender and age-related differences in some nonlinear components of HRV [4–6]. There also seemed to be a significant difference between day and night observation of HRV nonlinear indices using spectral and time domain methods [6].

Some authors suggested detecting and classifying arrhythmia based on nonlinear modeling of ECG [7, 8], predicting various cardiovascular diseases like ventricular tachycardia and congestive heart failure [9]. Chua et al. [10] introduced a method to extract bispectral entropy from HR signals by employing Higher Order Spectra techniques. In their study, HOS features from HR signals were used to differentiate between a normal heartbeat and seven arrhythmia classes.

Based on nonlinear parameters there are methods developed for fetal heart rate classification [11], spotting heart rhythm patterns in patient with diabetes [12], and detecting ventricular fibrillation [13]; the fractal structure of heart rate variability was identified in obese children [14]; the concurrent linear and nonlinear analysis of normal and CAD-affected heart rate signals was conducted [15]. Schubert et al. applied a dimensional complexity measure to heart rate time series in two stress conditions: chronic stress reported by healthy subjects and acute stress to psychologically challenging speech task stressor [16]. Su et al. analyze the dynamics of the heart rate signal before, during, and after an epileptic event [17]. Some authors reported the association between the HRV correlation dimension of patients with various clinical disorders and their survival prognosis [18].

The advantages of one group of methods over another are not yet that obvious. Possibly, it is because of the yet unknown mechanisms of complex heart rate regulation leading to the changes in specific nonlinear parameters. There is only a prevailing opinion that nonlinear dynamics of heart rate is the result of joint effect of parasympathetic and sympathetic influences.

For example, the correlation dimension *D*_2_ gives the information about the number of independent functional components necessary to describe the underlying system and the degree of nonlinear coupling between these components. In biological systems, the higher the *D*_2_ is, the more degrees of freedom the system has and, therefore, the greater range of possible adaptive responses is. The correlation dimension is usually calculated through the correlation integral using the algorithm of Grassberger and Procaccia [19]. There has been a lot of research lately on dimension of heart rhythm. The authors focused on investigating dimension of normal sinus rhythm, including the effect of circadian rhythms, influence of autonomous nervous system, and rhythm after heart transplants and in different pathologic conditions [20–25].

The correlation integral is also used to calculate the correlation entropy *K*_2_. Meanwhile, the correlation entropy describes the behavior of a system in terms of randomness and quantifies information about underlying dynamics. It is a dynamic measure and represents the rate at which information needs to be created as the chaotic system evolves in time. Entropy refers to system randomness, regularity, and predictability and allows systems to be quantified by rate of information loss or generation. Traditionally, *K*_2_ has been much less popular compared to *D*_2_ as a discriminating statistic in analyzing time series in practice. However, *K*_2_ has a significant and more relevant status, especially in the context of colored noise contamination, as indicated by many authors [26–28].

Nevertheless, there are still significant methodological problems of computation of nonlinear parameters, one of which is the choice of minimally acceptable sample size of RR intervals time series. Different criteria exist for minimal length of time series for analysis. For example, Tsonis criterion defines minimal length as *N* ≥ 10^2+0,4*D*_2_^, where *D*_2_ is assumed correlation dimension of reconstructed attractor [29]. According to other criteria, *N* > 10^(*D*_2_+2)/2^ [30], *N* ≥ 2^*D*_2_(*D*_2_+1)^ [31], or *N* ≥ 10^*D*_2_/2^ [32]. Finally, there is an opinion that the minimal series length depends not only on dimension parameters but also on process autocorrelation, and *N* ≥ 2*τ* ^*m*/2^ (where *τ* is autocorrelation time, i.e., number of steps till its first zero or minimum, and *m* is embedding dimension) [33].

To fulfill these requirements, the RR intervals' time series could be as long as 10000 intervals or even more, which, at the average heart rate of 70 per minute, requires more than 2 hours of ECG recording. In majority of publications, the data belongs to 24-hour monitoring of ECG; some researchers use time series of 500 to 1000 intervals without providing any rationale [15, 34–36]. In mass screenings and in some functional probes, such long recordings are not possible. The most preferable recording time at these situations is 5 minutes as recommended by Task Force of the European Society of Cardiology and the North American Society of Pacing and Electrophysiology [37].

Our goal was to develop the method to calculate the nonlinear parameters using 5-minute ECG recordings. We applied and tested our method in calculation of correlation dimension and correlation entropy. However, using the same approach, one could also calculate other nonlinear parameters.

## 2. Materials and Methods

**Figure 1:**
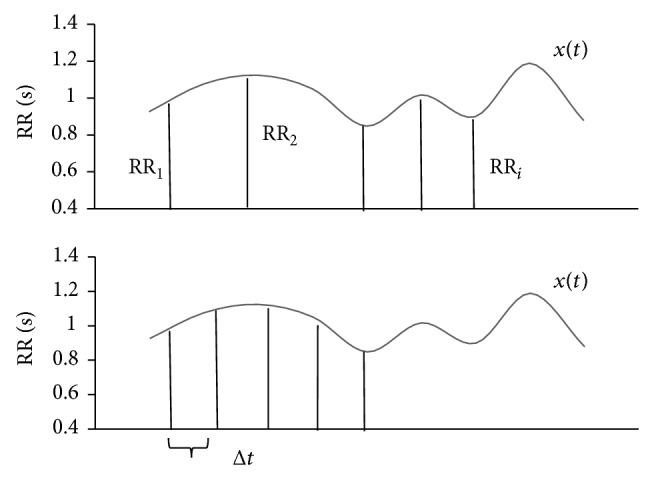
Resampling of RR series.

We analyzed 383 ECG recordings of practically healthy subjects aged 8 to 63. We sampled the ECG signals at a rate of 1,000 Hz and digitized them with a 16-bit analog-to-digital converter. The digitized signal was then averaged by shifting window of 32 ms to get rid of high-frequency noise. The modified Pan and Tompkins real-time QRS detection algorithm [38] was used to automatically detect R-waves and build RR interval series. According to its authors, this time-frequency algorithm has the reported correct detection rate of 99.3%. The original RR interval series were resampled by cubic spline interpolation *x*(*t*) using sampling time *dt* to obtain equidistantly sampled time series *x*_*i*_, *i* = 1,2,…, *N* (Figure 1).

To provide the rationale for parameters of the proposed method, we processed 38 sequences of RR intervals of persons aged 18–25 and 27 sequences of children aged 12-13 recorded for 5 minutes in rest. Quantization of the acquired continuous function *x*(*t*) at 5-minute interval of registration was done at different sampling rate *dt* of 250, 200, 125, and 100 ms.

We then computed the correlation dimension and entropy of these reprocessed time series using the algorithm proposed by Grassberger and Procaccia [19] with *r* = 15% from SDRR (standard deviation of RR intervals) [38].

Correlation dimension (*D*_2_) is a useful measure of self-similarity of a signal. According to the algorithm, correlation integral *C*(*N*, *m*, *r*) function is constructed first. The correlation integral, which counts the fraction of pairs (*X*_*i*_, *X*_*j*_) whose distance is smaller than *r*, is defined by

(1) $$\begin{array}{c} C\left(N,m,r\right)=\frac{1}{N\left(N-1\right)}\overset{N}{\underset{i=1}{\sum }}\overset{N}{\underset{j=i+1}{\sum }}H\left(r-\left|X_{i}-X_{j}\right|\right), \end{array}$$

where *X*_*i*_ and *X*_*j*_ indicate phase-space trajectory points, *N* is total amount of phase-space points, *H* is the Heaviside step function, *H*(*α*) = 0 if *α* < 0, and *H*(*α*) = 1 if *α* ≥ 0.

Correlation dimension is computed as

(2) $$\begin{array}{c} D_{2}=\underset{r\to 0}{\lim}\frac{\text{lg}\;C}{\text{lg}\;r}. \end{array}$$

*D*_2_ will have higher value if RR intervals vary more and vice versa.

Correlation entropy (*K*_2_) characterizes the probability of visiting space points by the trajectory of the examined system. If entropy reaches zero, the system becomes completely predictable. This is the case of regular processes. For truly random processes, the entropy is unlimitedly large. Entropy of “limited chaos” is positive but has the terminal value. The numerical entropy value quantitatively characterizes the level of system orderliness and is computed as

(3) $$\begin{array}{c} K_{2}=\underset{r\to o}{\ln}\frac{C\left(N,r,m\right)}{C\left(N,r,m+1\right)} \end{array}$$

with the same procedures used as for correlation dimension.

We used TISEAN software to compute these parameters. The given method was then tested in two models.

*The first model* included persons with different autonomic balancing of heart rate regulation. This balancing may be measured by HRV spectral characteristics. The amplitude of high-frequency (HF) component of HRV spectrum (ranged from 0,16 to 0,40 Hz) is considered to be related to vagal influence on heart rate [39]; the low frequency (LF) component at the same time is considered to be the market of sympathetic activity [40]. Correspondingly, the LF/HF ratio could be used to measure the level of autonomic balancing of heart rate regulation.

Using LF/HF, we selected three groups among the subjects included into research. The first group (*n* = 30) included the subjects with higher parasympathetic influence (LF/HF = 0,514 ± 0,041); the second group (*n* = 30) included subjects with balanced regulation (LF/HF = 1,130 ± 0,049); in the third group (*n* = 30), balancing was shifted towards sympathetic autonomous regulation (LF/HF = 3,474 ± 0,325).

We also tested* the second model* of age-related properties of nonlinear dynamics of heart rate. We used such a model because of certain knowledge on interrelation of neural regulation mechanisms and structural and functional development of organs and systems in different periods of human life. Relying on this knowledge, we could make assumptions about involvement of different parts of autonomous nervous system into the heart rate regulation and about mechanisms of aperiodic oscillations in HRV. We computed *D*_2_ and *K*_2_ in the following age groups: 8–13 (*n* = 34), 14–17 (*n* = 40), 18–21 (*n* = 21), 22–35 (*n* = 66), 36–55 (*n* = 50), and 55–63 (*n* = 17) years of age.

We used one-way ANOVA followed by Scheffé's test for multiple comparisons to compare various groups. Differences with *P* < 0.05 were considered to be statistically significant.

## 3. Results and Discussion

### 3.1. Rationale for the Choice of Computation Parameters

Since different sampling time produces time series with different sample size, we had to make sure how that influenced computation of nonlinear parameters and what should be embedding dimension *m* for phase-space reconstruction. To do that, we calculated correlation dimension of each time series using different embedding dimensions *m* and sampling time *dt*.

**Figure 2:**
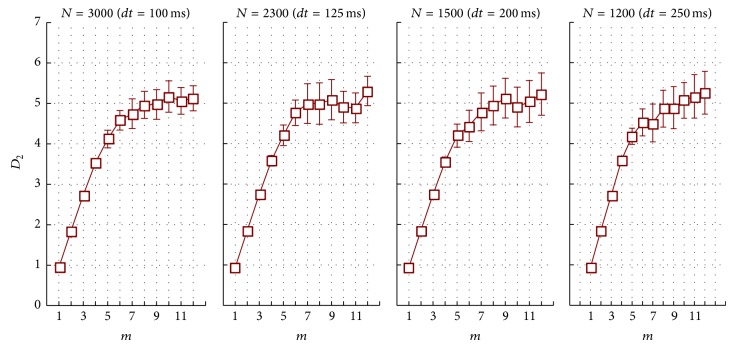
Dependence of correlation dimension on embedding dimension for phase-space reconstruction.

If the examined system contains* determined chaos*, the cross-plot of *D*_2_ against *m* is initially rising and then levels off.  Table 1 and Figure 2 show the results of such computations for adult ECG. It is obvious that, for all *dt* starting with *m* = 9, the changes in *D*_2_ become statistically insignificant and cross-plots level off.

Since physiological data tends to have significant intragroup variability and mean data does not always reflect individual patterns, we propose computing  *D*_2_ as the average of three measurements at *m* = 9,10,11.

The sample size *N* at different sampling time was from 1200 at *dt* = 250 ms to 3000 at *dt* = 100 ms. At this, on average, the values of correlation dimension were not statistically significant (Table 2); that is, sampling time did not influence the results. We got similar results analyzing ECG in children. Since the children's heart rhythm has different pattern compared to adults, we tested out assumptions in the second sample of junior schoolchildren aged 12-13. The differences in *D*_2_ means at different *dt* were not statistically significant (Table 2).

**Figure 3:**
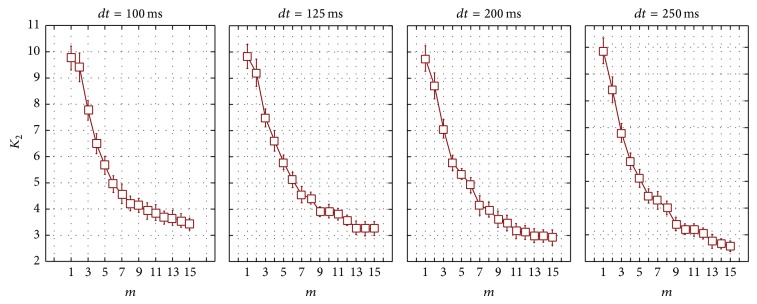
Dependence of correlation entropy on embedding dimension for phase-space reconstruction.

We also had to provide the rationale for selection of the same parameters for computation of correlation entropy *K*_2_. As obvious from Table 3 and Figure 3, its values decrease with the increase of *m* and then start to level off when embedding dimension *m* = 9 at all *dt*.

Table 4 displays the means of correlation entropy at different *dt*. We can see from this data that, at *dt* = 200 ms, *K*_2_ is statistically significantly reduced. Apparently, it is due to the smaller sample size (*N* ≈ 1500 at *dt* = 200 ms). The largest sample size is at *dt* = 100 ms, at this  *N* ≈ 3000, which is the closest to the specified requirements. That is why we considered it  reasonable to compute *K*_2_ the same way as *D*_2_ at *dt* = 100 ms.

### 3.2. Nonlinear HRV in Persons with Different Balancing of Autonomous Nervous System

Table 5 presents the results of computing correlation dimension and correlation entropy in groups with different balancing of activity of sympathetic and parasympathetic autonomous nervous systems. We see that when autonomous balancing shifts towards sympathetic regulation, the correlation dimension and entropy decrease; Group 3 has even statistically significant differences from Group 2.

The analysis of age-related differences indicated that the most complex heart rate dynamics was in junior and adolescent age groups of 8–13 and 14–17 years of age (Table 6). In comparison to 14–17-year olds, the age group of 18–21 had statistically lower values of *D*_2_ and *K*_2_.

Starting with age group of 22–35, the heart rate dynamics becomes simpler; the rhythm becomes less “chaotic” and more regular due to increased sympathetic activity. It is known that, starting with this age, the variation of RR intervals decreases; that is, the sympathetic nervous system starts to exercise its stabilizing effect. These processes are even more expressed in the age group of 55 and older, which is characterized by the lowest values of nonlinear parameters, that is, displaying the most regular rhythm without significant “chaotic” modulations.

Thus, our research provides the rationale for computation method of correlation dimension and correlation entropy using short (5-minute-long) HRV segments, which is especially important for mass screening outside the lab conditions. For this, the 5-minute RR intervals time series is presented as continuous function *x*(*t*). This function is then transformed into a new discrete time series through quantization with the sampling time *dt* = 100 ms. Then, the phase trajectory is reconstructed using the time delay method with time delay *τ* selected as the first minimum of autocorrelation function. The correlation dimension and correlation entropy are then computed using the presented algorithm with parameters *r* = 15% of SDRR averaged for *m* = 9,10,11.

We proved the rationale for the proposed method using it to measure autonomous balancing. According to acquired results, the autonomous nervous system significantly affects heart rate irregularity. The largest complexity and “chaotic character” of heart rhythm are  observed in persons with predominant influence of parasympathetic branch of autonomous nervous system. The shift towards sympathetic regulation brings, on contrary, the regularity and makes the heart rhythm less “chaotic” and simpler in dynamics. Nevertheless, the outcome is not simply the sum of these influences but the result of simultaneous activation of both sympathetic and parasympathetic branches of autonomous nervous system. We confirmed this fact analyzing nonlinear parameters in different age groups. It is known that, by the age of ten, the cholinergic influence becomes predominant, but adrenergic mechanisms still play a role. Adrenergic influences affect different pieces of acetylcholine metabolism, defining the development speed of cholinergic regulation of heart. The joint effect of both sympathetic and parasympathetic effects causes changes in heart rhythm, including its irregular component. According to our data, the childhood and adolescence are characterized by the most complex heart rate dynamics, when parasympathetic nervous system plays a dominant role against the background of elevated tonus of sympathetic branch. Similar results were acquired in the research of autonomous nervous system reaction to cold pressure test as well as in the experiment with intravenous administration of different doses of noradrenaline, accompanied by the reduction of heart rate and elevation of blood pressure [41, 42].

## 4. Conclusion

Even though the nonlinear methods are currently widely used to analyze the heart rate in different functional conditions and diseases, the computation methods used are not standardized as it has been done for linear parameters [37]. It would be convenient to use 5-minute ECG recordings as the basic methodology. Our proposed method of computation may become the basis for such standardization. We displayed its suitability for computation of *D*_2_ and *K*_2_, but there is a need for further research to show its application to calculation of other nonlinear parameters.

## Figures and Tables

**Table 1:** Means (standard deviations) of correlation dimension using different sampling times.

Sampling time	*dt* = 100 ms	*dt* = 125 ms	*dt* = 200 ms	*dt* = 250 ms

Embedding dimension	Mean (SD)	Mean (SD)	Mean (SD)	Mean (SD)

*m* = 1	0,943 (0,037)	0,943 (0,037)	0,939 (0,037)	0,941 (0,037)
*m* = 2	1,845 (0,111)^*^	1,844 (0,105)^*^	1,843 (0,099)^*^	1,841 (0,105)^*^
*m* = 3	2,66 (0,277)^*^	2,69 (0,253)^*^	2,708 (0,222)^*^	2,724 (0,228)^*^
*m* = 4	3,448 (0,425)^*^	3,525 (0,29)^*^	3,511 (0,284)^*^	3,499 (0,308)^*^
*m* = 5	4,14 (0,462)^*^	4,244 (0,512)^*^	4,235 (0,462)^*^	4,178 (0,493)^*^
*m* = 6	4,57 (0,561)^*^	4,736 (0,518)^*^	4,628 (0,752)^*^	4,645 (0,684)^*^
*m* = 7	4,857 (0,888)^*^	4,992 (0,777)^*^	5,101 (0,808)^*^	4,981 (0,783)^*^
*m* = 8	4,992 (0,771)^*^	5,177 (0,992)^*^	5,163 (0,992)^*^	5,321 (0,875)^*^
*m* = 9	4,922 (0,814)	5,264 (0,925)	5,193 (0,986)	5,304 (1,023)
*m* = 10	4,996 (0,083)	5,364 (0,931)	5,412 (1,054)	5,637 (1,147)
*m* = 11	5,051 (0,863)	5,451 (0,888)	5,62 (1,091)	5,575 (1,042)
*m* = 12	5,133 (0,814)	5,631 (0,826)	5,717 (0,962)	5,732 (1,153)
*m* = 13	5,18 (0,826)	5,741 (0,974)	5,889 (1,103)	5,847 (1,251)

^*^Statistically significant changes with *m* from 1 to 13 (Scheffé's test, *P* < 0.05).

**Table 2:** Means (standard deviations) of correlation dimension at different sampling times of ECG in adults and children.

Age group	Sampling time				ANOVA test
	*dt* = 100 ms	*dt* = 125 ms	*dt* = 200 ms	*dt* = 250 ms	
	Mean (SD)	Mean (SD)	Mean (SD)	Mean (SD)	
Adults	5,069 (0,815)	4,959 (0,861)	5,041 (1,132)	5,062 (1,142)	n.s.
Children	5,757 (0,784)	5,814 (0,821)	5,851 (0,956)	5,745 (0,748)	n.s.

**Table 3:** Means (standard deviations) of correlation entropy using different sampling times.

Sampling time	*dt* = 100 ms	*dt* = 125 ms	*dt* = 200 ms	*dt* = 250 ms

Embedding dimension	Mean (SD)	Mean (SD)	Mean (SD)	Mean (SD)

*m* = 1	9,764 (1,123)	9,829 (1,139)	9,751 (1,202)	9,868 (1,185)
*m* = 2	9,407 (1,360)	9,204 (1,291)^*^	8,709 (1,232)^*^	8,418 (1,183)^*^
*m* = 3	7,766 (0,930)^*^	7,486 (0,852)^*^	7,066 (0,861)^*^	6,806 (0,877)^*^
*m* = 4	6,493 (0,941)^*^	6,612 (0,975)^*^	5,771 (0,717)^*^	5,750 (0,758)^*^
*m* = 5	5,664 (0,846)^*^	5,788 (0,701)^*^	5,346 (0,514)	5,109 (0,829)^*^
*m* = 6	4,951 (0,790)^*^	5,114 (0,719)^*^	4,917 (0,714)	4,448 (0,660)^*^
*m* = 7	4,580 (0,927)	4,553 (0,770)^*^	4,146 (0,917)^*^	4,285 (0,828)
*m* = 8	4,215 (0,691)	4,423 (0,570)	3,983 (0,697)	4,006 (0,626)
*m* = 9	4,140 (0,663)	3,905 (0,458)^*^	3,617 (0,776)	3,402 (0,590)^*^
*m* = 10	3,927 (0,783)	3,926 (0,645)	3,477 (0,739)	3,210 (0,492)
*m* = 11	3,867 (0,749)	3,809 (0,548)	3,167 (0,721)	3,178 (0,581)
*m* = 12	3,674 (0,622)	3,579 (0,492)	3,111 (0,651)	3,050 (0,505)
*m* = 13	3,665 (0,708)	3,296 (0,634)	2,993 (0,661)	2,757 (0,642)
*m* = 14	3,565 (0,645)	3,255 (0,676)	2,977 (0,592)	2,665 (0,470)
*m* = 15	3,428 (0,552)	3,262 (0,663)	2,923 (0,719)	2,574 (0,492)

^*^Statistically significant changes with *m* from 1 to 13 (Scheffé's test, *P* < 0.05).

**Table 4:** Means (standard deviations) of correlation entropy at different sampling times of ECG.

Parameter	Sampling time			
	*dt* = 100 ms	*dt* = 125 ms	*dt* = 200 ms	*dt* = 250 ms
	Mean (SD)	Mean (SD)	Mean (SD)	Mean (SD)
*K*_2_	3,915 (0,428)	3,942 (0,428)	3,534 (0,418)^*^	3,459 (0,362)

^*^Statistically significant changes compared to previous *dt* (Scheffé's test, *P* < 0.05).

**Table 5:** Nonlinear parameters of HRV in groups with different balancing of activity of autonomous nervous system.

	Group 1	Group 2	Group 3
*D*_2_	5,782 (1,068)	5,671 (1,106)	5,063 (1,342)^*^
*K*_2_	4,556 (0,805)	4,506 (0,733)	4,110 (0,854)^*^

^*^Statistically significant differences from the previous group (Scheffé's test, *P* < 0.05).

**Table 6:** Means (standard deviations) of HRV nonlinear parameters in different age groups.

Age groups, years of age						
	8–13	14–17	18–21	22–35	36–55	Older than 55
	Mean (SD)	Mean (SD)	Mean (SD)	Mean (SD)	Mean (SD)	Mean (SD)
*D*_2_	5,96 (0,70)	6,06 (0,63)	5,11 (0,73)^*^	4,92 (0,89)	4,37 (0,64)^*^	3,78 (1,65)^*^
*K*_2_	4,37 (0,52)	4,72 (0,51)^*^	3,94 (0,50)^*^	3,85 (0,57)	3,60 (0,64)^*^	3,38 (0,95)^*^

^*^Statistically significant differences from the previous group (Scheffé's test, *P* < 0.05).

## References

[1] Schumacher A. (2004). Linear and nonlinear approaches to the analysis of R-R interval variability. *Biological Research For Nursing*.

[2] Buccelletti F., Bocci M. G., Gilardi E. (2012). Linear and nonlinear heart rate variability indexes in clinical practice. *Computational and Mathematical Methods in Medicine*.

[3] Narin A., Isler Y., Ozer M. (2014). Investigating the performance improvement of HRV Indices in CHF using feature selection methods based on backward elimination and statistical significance. *Computers in Biology and Medicine*.

[4] Ryan S. M., Goldberger A. L., Pincus S. M., Mietus J., Lipsitz L. A. (1994). Gender- and age-related differences in heart rate dynamics: are women more complex than men?. *Journal of the American College of Cardiology*.

[5] Yamasaki Y., Kodama M., Matsuhisa M. (1996). Diurnal heart rate variability in healthy subjects: effects of aging and sex difference. *American Journal of Physiology—Heart and Circulatory Physiology*.

[6] Beckers F., Verheyden B., Aubert A. E. (2006). Aging and nonlinear heart rate control in a healthy population. *American Journal of Physiology: Heart and Circulatory Physiology*.

[7] Owis M. I., Abou-Zied A. H., Youssef A.-B. M., Kadah Y. M. (2002). Study of features based on nonlinear dynamical modeling in ECG arrhythmia detection and classification. *IEEE Transactions on Biomedical Engineering*.

[8] No Y. S., Kapluk C., Krishnan S. M. (2000). Arrhythmia detection and recognition in ECG signals using nonlinear techniques. *Annals of Biomedical Engineering*.

[9] Cohen M., Hudson D. L., Deedwania P. C. (1996). Heart rate variability and cardiovascular mortality. *IEEE Engineering in Medicine and Biology Magazine*.

[10] Chua K. C., Chandran V., Acharya U. R., Lim C. M. (2008). Computer-based analysis of cardiac state using entropies, recurrence plots and Poincare geometry. *Journal of Medical Engineering and Technology*.

[11] Spilka J., Chudáček V., Koucký M. (2012). Using nonlinear features for fetal heart rate classification. *Biomedical Signal Processing and Control*.

[12] Acharya U. R., Faust O., Kadri N. A., Suri J. S., Yu W. (2013). Automated identification of normal and diabetes heart rate signals using nonlinear measures. *Computers in Biology and Medicine*.

[13] Roopaei M., Boostani R., Sarvestani R. R., Taghavi M. A., Azimifar Z. (2010). Chaotic based reconstructed phase space features for detecting ventricular fibrillation. *Biomedical Signal Processing and Control*.

[14] Vanderlei L. C. M., Pastre C. M., Júnior I. F. F., de Godoy M. F. (2010). Fractal correlation of heart rate variability in obese children. *Autonomic Neuroscience: Basic and Clinical*.

[15] Acharyaa U. R., Fausta O., Sreec V. (2014). Linear and nonlinear analysis of normal and CAD-affected heart rate signals. *Сomputer Methods and Programs in Biomedicine*.

[16] Schubert C., Lambertz M., Nelesen R. A., Bardwell W., Choi J.-B., Dimsdale J. E. (2009). Effects of stress on heart rate complexity. A comparison between short-term and chronic stress. *Biological Psychology*.

[17] Su Z.-Y., Wu T., Yang P.-H., Wang Y.-T. (2008). Dynamic analysis of heartbeat rate signals of epileptics using multidimensional phase space reconstruction approach. *Physica A: Statistical Mechanics and Its Applications*.

[18] Almoznino-Sarafian D., Sarafian G., Zyssman I. (2009). Application of HRV-CD for estimation of life expectancy in various clinical disorders. *European Journal of Internal Medicine*.

[19] Grassberger P., Procaccia I. (1983). Characterization of strange attractors. *Physical Review Letters*.

[20] Destexhe A., Sepulchre J. A., Babloyantz A. (1988). A comparative study of the experimental quantification of deterministic chaos. *Physics Letters A*.

[21] Ganz R. E., Faustmann P. M. (1993). Central cardio-autonomic disorganization in interictal states of epilepsy. *International Journal of Neuroscience*.

[22] Hoekstra B. P. T., Diks C. G. H., Allessie M. A. (1995). Nonlinear analysis of epicardial electrograms of electrically induced atrial fibrillation in man. *Journal of Cardiovascular Electrophysiology*.

[23] Moore R. K. G., Groves D., Kearney M. T. (2004). HRV spectral power and mortality in chronic heart failure (CHF): 5 year results of the UK heart study. *Heart A*.

[24] Huikuri H. V., Perkiömäki J. S., Maestri R., Pinna G. D. (2009). Clinical impact of evaluation of cardiovascular control by novel methods of heart rate dynamics. *Philosophical Transactions of the Royal Society A: Mathematical, Physical and Engineering Sciences*.

[25] Huikuri H. V., Mäkikallio T. H., Peng C. K., Goldberger A. L., Hintze U., Møller M. (2000). Fractal correlation properties of R-R interval dynamics and mortality in patients with depressed left ventricular function after an acute myocardial infarction. *Circulation*.

[26] Szépfalusy P., Györgyi G. (1986). Entropy decay as a measure of stochasticity in chaotic systems. *Physical Review A*.

[27] Redaelli S., Plewczyński D., Macek W. M. (2002). Influence of colored noise on chaotic systems. *Physical Review E*.

[28] Urbanowicz K., Holyst J. A. (2003). Noise level estimation of time series using coarse grained entropy. *Physical Review E*.

[29] Tsonis A. (1992). *Chaos: From Theory to Applications*.

[30] Kantz H., Schreiber T. (1995). Dimension estimates and physiological data. *Chaos*.

[31] Essex C., Nerenberg M. A. H. (1996). Comment on ‘deterministic chaos: the science and the fiction’. *Proceedings of the Royal Society of London A*.

[32] Ruelle D. (1990). Deterministic chaos: the science and the fiction. *Proceedings of the Royal Society of London Series A: Mathematical, Physical and Engineering Sciences*.

[33] Theiler J. (1986). Spurious dimension from correlation algorithms applied to limited time-series data. *Physical Review A*.

[34] Vanderlei L. C. M., Pastre C. M., Júnior I. F. F., de Godoy M. F. (2010). Fractal correlation of heart rate variability in obese children. *Autonomic Neuroscience: Basic and Clinical*.

[35] Jovic A., Bogunovic N. (2011). Electrocardiogram analysis using a combination of statistical, geometric, and nonlinear heart rate variability features. *Artificial Intelligence in Medicine*.

[36] Graff B., Szyndler A., Czechowicz K. (2013). Relationship between heart rate variability, blood pressure and arterial wall properties during air and oxygen breathing in healthy subjects. *Autonomic Neuroscience: Basic and Clinical*.

[37] Task Force of the European Society of Cardiology and the North American Society of Pacing and Electrophysiology (1996). Heart rate variability. Standarts of Mesurement, physiological interpretation and clinical use. *Circulation*.

[38] Pan J., Tompkins W. J. (1985). A real-time QRS detection algorithm. *IEEE Transactions on Biomedical Engineering*.

[39] Jiang W., Hayano J., Coleman E. R. (1993). Relation of cardiovascular responses to mental stress and cardiac vagal activity in coronary artery disease. *The American Journal of Cardiology*.

[40] Pagani M., Mazzuero G., Ferrari A. (1991). Sympatovagal interaction during mental stress. A study using spectral analysis of heart rate variability in healthy control subjects and patients with a prior myocardial infarction. *Circulation*.

[41] Tulppo M. P., Kiviniemi A. M., Hautala A. J. (2005). Physiological background of the loss of fractal heart rate dynamics. *Circulation*.

[42] Tulppo M. P., Mäkikallio T. H., Seppänen T., Airaksinen J. K. E., Huikuri H. V. (1998). Heart rate dynamics during accentuated sympathovagal interaction. *American Journal of Physiology—Heart and Circulatory Physiology*.

